# Draft Genome of the Common Snapping Turtle, *Chelydra serpentina*, a Model for Phenotypic Plasticity in Reptiles

**DOI:** 10.1534/g3.120.401440

**Published:** 2020-09-30

**Authors:** Debojyoti Das, Sunil Kumar Singh, Jacob Bierstedt, Alyssa Erickson, Gina L. J. Galli, Dane A. Crossley, Turk Rhen

**Affiliations:** * Department of Biology, University of North Dakota, Grand Forks, North Dakota 58202; † Division of Cardiovascular Sciences, School of Medical Sciences, University of Manchester, Manchester M13 9NT, UK; ‡ Department of Biological Sciences, University of North Texas, Denton, Texas 76203

**Keywords:** Snapping turtle, *Chelydra serpentina*, genome assembly, genome annotation, phenotypic plasticity

## Abstract

Turtles are iconic reptiles that inhabit a range of ecosystems from oceans to deserts and climates from the tropics to northern temperate regions. Yet, we have little understanding of the genetic adaptations that allow turtles to survive and reproduce in such diverse environments. Common snapping turtles, *Chelydra serpentina*, are an ideal model species for studying adaptation to climate because they are widely distributed from tropical to northern temperate zones in North America. They are also easy to maintain and breed in captivity and produce large clutch sizes, which makes them amenable to quantitative genetic and molecular genetic studies of traits like temperature-dependent sex determination. We therefore established a captive breeding colony and sequenced DNA from one female using both short and long reads. After trimming and filtering, we had 209.51Gb of Illumina reads, 25.72Gb of PacBio reads, and 21.72 Gb of Nanopore reads. The assembled genome was 2.258 Gb in size and had 13,224 scaffolds with an N50 of 5.59Mb. The longest scaffold was 27.24Mb. BUSCO analysis revealed 97.4% of core vertebrate genes in the genome. We identified 3.27 million SNPs in the reference turtle, which indicates a relatively high level of individual heterozygosity. We assembled the transcriptome using RNA-Seq data and used gene prediction software to produce 22,812 models of protein coding genes. The quality and contiguity of the snapping turtle genome is similar to or better than most published reptile genomes. The genome and genetic variants identified here provide a foundation for future studies of adaptation to climate.

Turtles are a monophyletic group of reptiles recognized by their shell, a unique adaptation that makes them an iconic animal (Lyson *et al.* 2013). There are 356 turtle species divided between two suborders. The Cryptodira or hidden-necked turtles include 263 species, while the Pleurodira or side-necked turtles include 93 species (Rhodin *et al.* 2017). Phylogenomic analysis of 26 species across 14 known families has produced a well-resolved tree showing relationships among 11 cryptodiran and 3 pleurodiran families (Shaffer *et al.* 2017). Since their origin 220 million years ago, turtles have evolved the ability to inhabit a wide array of aquatic and terrestrial ecosystems, ranging from oceans to deserts. Yet, turtles are one of the most threatened vertebrate groups. Roughly 60% of turtle species on the IUCN Red List (2017) are considered vulnerable, endangered, or critically endangered (Stanford *et al.* 2018). Habitat destruction, overharvest, and international trade are the main causes of population decline (Böhm *et al.* 2013, Stanford *et al.* 2018).

Climate change is another major concern, especially for turtles with temperature-dependent sex determination (TSD) (Mitchell and Janzen 2010, Santidrián Tomillo *et al.* 2015, Hays *et al.* 2017). Although incubation studies have only been carried out on a subset of species, most turtles examined (81%) exhibit TSD (Ewert *et al.* 2004). Phylogenetic analyses indicate that TSD is the ancestral mode of sex determination and that genotypic sex determination evolved independently several times (Janzen and Krenz 2004, Valenzuela and Adams 2011, Pokorná and Kratochvil 2016). In addition to its effect on the gonads, incubation temperature has a significant impact on growth, physiology, and behavior in turtles and other reptiles (Rhen and Lang 2004, Noble *et al.* 2018, While *et al.* 2018, Singh *et al.* 2020).

Temperature effects are a specific example of a broader phenomenon called phenotypic plasticity in which environmental factors alter phenotype (Via and Lande 1985, Scheiner 1993, Agrawal 2001, Angilletta 2009, Warner *et al.* 2018). Organisms can also maintain phenotypic stability in the face of variable environments. Physiologists call this homeostasis while developmental biologists call it canalization. Although plasticity and stability appear to be distinct strategies for dealing with environmental variation, they actually represent ends of a continuum of potential responses. Plasticity/stability often has a genetic basis with different individuals being more or less responsive to environmental influences. We must decipher genome-environment interactions to understand the role plasticity/stability plays in allowing turtles to survive and reproduce in diverse climates from the tropics to temperate regions.

Genomic resources will facilitate research on the evolution of phenotypic plasticity, homeostasis, and developmental canalization in turtles. To date, genomes from six turtle species in five families have been sequenced (Shaffer *et al.* 2013, Wang *et al.* 2013, Tollis *et al.* 2017, Cao *et al.* 2019, https://www.ncbi.nlm.nih.gov/bioproject/PRJNA415469/], but each of these species lives and reproduces in a much narrower range of climates than the common snapping turtle (*Chelydra serpentina*). Here we assemble and annotate the first draft genome for the snapping turtle, which is the most widespread and abundant species in the family Chelydridae.

The contiguity and completeness of the snapping turtle genome is similar to or better than other reptiles and is adequate for reuse in functional and comparative genomic studies. Several characteristics make the snapping turtle a good model for turtle biology. This species is one of the most extensively studied turtles (Steyermark *et al.* 2008, While *et al.* 2018), providing a wealth of baseline information for genetic, genomic, epigenomic, and transcriptomic analyses of cell and developmental biology, physiology, behavior, ecology and evolution. This species produces large clutches (30-95 eggs/clutch) and is easy to breed and rear in captivity, making genetic studies feasible. We therefore established a captive breeding colony to study phenotypic variation in TSD (Janzen 1992, Rhen and Lang 1998, Ewert *et al.* 2005, Rhen *et al.* 2015, Schroeder *et al.* 2016). Controlled breeding reveals that variation in TSD within populations is highly heritable and that population differences in sex ratio at warm incubation temperatures are also heritable (K. Hilliard and T. Rhen, unpublished results). Yet, population differences in sex ratio at cool incubation temperatures are due to genetic dominance and/or non-genetic maternal effects, illustrating genome-environment interactions (K. Hilliard and T. Rhen, unpublished results). These findings provide a solid foundation for genome-wide association studies to identify specific loci that influence thermosensitivity (Schroeder *et al.* 2016).

The genome will also be useful for studying other ecologically important traits and characterizing population genomic variation. Such studies will provide insight into genetic adaptation to climate because snapping turtles range from tropical to northern temperate zones. For example, snapping turtles display counter-gradient variation in developmental rate with latitude: northern alleles speed embryonic developmental rate to counteract the impact of cooler soil temperatures at higher latitudes (K. Hilliard and T. Rhen, unpublished results). Another remarkable trait is their ability to tolerate hypoxic conditions. Eggs buried underground periodically experience low oxygen conditions (*e.g.*, when soil is saturated with water after heavy rains). Hypoxia during embryogenesis programs subsequent performance in low oxygen environments: cardiomyocytes from juvenile snapping turtles exposed to hypoxia as embryos have enhanced myofilament Ca^2+^-sensitivity and ability to curb production of reactive oxygen species when compared to juveniles exposed to normoxic conditions as embryos (Ruhr *et al.* 2019). Such findings have broader implications for understanding cardiac hypoxia tolerance/susceptibility across vertebrates: *i.e.*, most human diseases of the heart are due to insufficient oxygen supply. A contiguous, well-annotated genome is critical for epigenomic studies of developmentally plastic responses to temperature and oxygen levels as well as other abiotic factors and ecological interactions. For instance, future studies will correlate genome-wide patterns of DNA methylation with transcriptome-wide patterns of gene expression in hearts of juvenile turtles exposed to hypoxic conditions as embryos.

The genome will also be valuable for comparative studies with other Chelydridae, which are listed as vulnerable on the ICUN Red List (2017): *Macrochelys temminckii* in North America, *Chelydra rossignoni* in Central America, and *Chelydra acutirostris* in South America. Finally, we expect this draft will serve as a template for refinement and improvement of the snapping turtle genome assembly.

## Materials and Methods

### Animal husbandry

Adult snapping turtles were captured by hand, with baited hoop nets, and during fish surveys in the state of Minnesota (MN) and transported to the University of North Dakota (UND) to establish a captive breeding colony for genetic analysis of TSD. Turtles were collected across the state of MN from the Canadian border in the north to the Iowa border in the south, which spans a 5° latitudinal range. Turtles in the colony are housed year-round in the animal quarters at UND in conditions that mimic seasonal changes in photoperiod and water temperature in MN.

Two rooms are set up with seven stock tanks per room (14 total tanks). Turtles are held in 1136-liter stock tanks (2.3 m long × 1.9 m wide × 1.6 m deep) filled with roughly 850 liters of water. One male is housed with 3 or 4 females per tank in a paternal half-sib, maternal full-sib mating design (K. Hilliard and T. Rhen, unpublished results). These tanks are 8x as long, 3.5x as wide, and 5x as deep as the average adult snapping turtle. Snapping turtles inhabit streams of similar width and depth. This provides room for the largest turtles to swim freely. Water flows continuously through tanks at a velocity similar to moving water that turtles experience naturally.

Water efflux from seven tanks passes through a multi-step filtration, sterilization, and temperature control system. The first step is mechanical filtration of solid waste as water flows into a ProfiDrum Eco 45/40 Rotary Drum Filter (RDF), which filters particles larger than 70 microns. In the second step, water passes from the RDF into a Sweetwater Low-Space Bioreactor seeded with bacteria that degrade nitrogenous wastes. In the third step, filtered water is pumped through an Emperor Aquatics SMART High Output UV Sterilizer to kill potential pathogens. In the final step, filtered and sterilized water flows through Aqua Logic Multi-Temp Chillers to control water temperature and is fed back into stock tanks. A constant turnover of 850 liters per day of fresh, de-chlorinated water is fed into the system with excess dirty water flowing out of the system into a floor drain. Water is re-circulated through the system at a rate of 2 complete water changes/tank/hour.

### Sample collection and DNA sequencing

We extracted DNA from one adult female snapping turtle in our breeding colony. This female was captured by the MN Department of Natural Resources during a fish survey of Mons Lake in central Minnesota, USA (45.9274° N, 94.7078° W) in June of 2010. We removed the female from her tank during mid-winter (water and body temperature ∼3°). Skin on the dorsum of the neck was sterilized with 70% ethanol and blood was drawn from the subcarapacial vein as described by Moon and Hernandez Foerster (2001). Whole blood was transferred to a microfuge tube and kept on ice until genomic DNA was extracted using a genomic-tip 100/G kit (Qiagen).

DNA quantity was measured using Quanti-iT PicoGreen dsDNA kit and a Qubit fluorometer. DNA purity was assessed via measurement of absorbance (A230/A260 and A260/280 ratios) on a Nanodrop spectrophotometer. All DNA samples had A260/A280 ratios between 1.8 and 2.0 and A260/230 ratios between 2.0 and 2.3. DNA integrity was examined via 0.8% agarose gel electrophoresis and/or the Agilent TapeStation. Sample DNA was much longer than the 23 kb marker from a HindIII digested Lambda phage ladder when run on agarose gels. Sample DNA was also longer than the 48.5 kb marker when run on the Agilent TapeStation.

High molecular weight genomic DNA was shipped on dry ice to the High Throughput Genomics Core Facility at the Huntsman Cancer Institute, University of Utah. The facility used the Illumina TruSeq DNA PCR-Free Sample Prep protocol to make a short insert (∼200 bp) library for 2 × 125 cycle paired end sequencing. The facility also used the Sage Science ELF electrophoresis system and Nextera MatePair Sample Preparation Kit to make two long insert (∼5.2kb and 10kb) libraries for 2 × 125 cycle paired end sequencing. Sequencing on the Illumina HiSeq 2000 instrument produced a total of 197.58Gb of raw data (Table 1). To increase sequencing depth and augment the diversity of long insert sizes, we sent high molecular weight genomic DNA to the Sequencing Center at Brigham Young University. Two additional mate pair libraries were prepared with average insert sizes of 3kb and 20kb for 2 × 125 cycle paired end sequencing on the Illumina HiSeq 2500. This produced another 173.08Gb of raw data (Table 1). After trimming and filtering for read quality and adapters, there was a total of 236.89Gb of short read data (Table 1). Using the size of the draft genome (2.258Gb), average coverage with fully processed Illumina reads was approximately 104.9x.

**Table 1:** Summary of whole genome shotgun sequence data for *Chelydra serpentina*

Platform	Seq Center	Library Type	Nominal insert size	Lane or Cell	Raw Reads	Filtered Reads	Mean read length	Bases (Gb)
HiSeq 2000	HCI	Paired-end	200 bp	7	157628596	148173891	124	18.37356248
HiSeq 2000	HCI	Mate-pair	5.2 kb	7	169828370	186726281	124	23.15405884
HiSeq 2000	HCI	Mate-pair	10 kb	7	217064820	238907655	124	29.62454922
HiSeq 2000	HCI	Paired-end	200 bp	1	513299776	488731386	124	60.60269186
HiSeq 2000	HCI	Paired-end	200 bp	8	522818714	480522479	124	59.5847874
				**total =**	**1580640276**		**total =**	**191.3396498**
PacBio Sequel	RTL	SMRT	30 kb	1	505167	504425		4.62472795
PacBio Sequel	RTL	SMRT	30 kb	2	728665	727435		5.42416282
PacBio Sequel	RTL	SMRT	30 kb	3	329474	329001		2.226995261
PacBio Sequel	RTL	SMRT	30 kb	4	493154	492682		4.032488331
PacBio Sequel	RTL	SMRT	30 kb	5	447251	446685		3.649041827
PacBio Sequel	RTL	SMRT	30 kb	6	687664	686769		5.512641628
				**total =**	**3191375**			**25.47005782**
HiSeq 2500	BYU	Mate-pair	3kb	1	545122952	255825287	89	22.76845054
HiSeq 2500	BYU	Mate-pair	3kb	2	545477596	255974272	89	22.78171021
HiSeq 2500	BYU	Mate-pair	20kb	1	294039334	116881537	90	10.51933833
				**total =**	**1384639882**		**total =**	**45.55016075**
Oxford	UND	Nanopore	N/A	Maxwell	560869	N/A	N/A	5.618462972
Oxford	UND	Nanopore	N/A	PC	391059	N/A	N/A	4.223690427
Oxford	UND	Nanopore	N/A	PC-SRE1	594707	N/A	N/A	3.521567316
Oxford	UND	Nanopore	N/A	PC-SRE2	644389	N/A	N/A	3.259168727
Oxford	UND	Nanopore	N/A	PC-SRE3	522053	N/A	N/A	5.103403101
				**total =**	**2713077**		**total =**	**21.726292543**

We also sent high molecular weight DNA on dry ice to RTL Genomics (Lubbock Texas) for long read sequencing. The facility prepared a PacBio SMRT (Single molecule, real time) library with Sequel chemistry and sequenced the library on 6 SMRT cells. PacBio sequencing produced a total of 25.72Gb of data, with the longest reads ranging from 73.6kb to 100.6kb (Table 1). Average coverage with PacBio long reads was approximately 11.4x.

Finally, we sequenced high molecular weight DNA using the Oxford Nanopore GridION X5 system in the Genomics Core at UND. We isolated fresh DNA from whole blood of the reference turtle using three methods: the Maxwell automated nucleic acid extraction system, phenol-chloroform extraction, and phenol-chloroform extraction with size selection via the Circulomics Short Read Eliminator Kit. Libraries were made using the Ligation Sequencing Kit (SQK-LSK109) and ran on version R9.4.1 flow cells in 1D, high accuracy mode. The library prepared with DNA from the Maxwell system was sequenced on one flow cell, phenol-chloroform extracted DNA was sequenced on one flow cell, while phenol-chloroform extracted and size selected DNA was sequenced on three flow cells. Nanopore sequencing produced a total of 21.72 Gb of data (Table 1), with the longest reads ranging from 180.5 kb to 273.8 kb. Average coverage with Nanopore long reads was approximately 9.6x.

### Short and long read quality control

**Figure 1:**
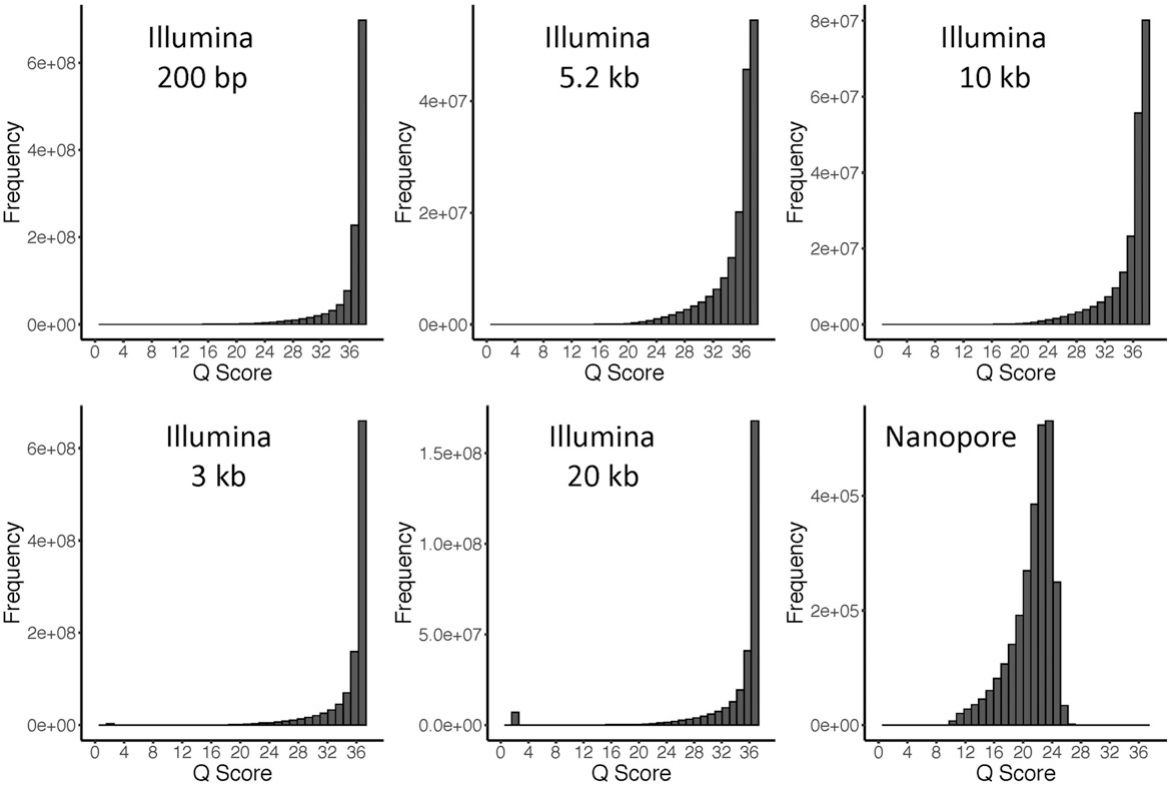
Histograms showing the distribution of raw read quality scores for Illumina and Nanopore libraries. The 200 bp, 5.2 kb, and 10 kb libraries were prepared and sequenced at Huntsman Cancer Institute, University of Utah. The 3 kb and 20 kb libraries were prepared and sequenced at Brigham Young University. The Nanopore libraries were prepared and sequenced at the University of North Dakota.

Raw quality scores for reads from Illumina libraries and the Nanopore libraries are shown in Figure 1. We examined read quality using the FastQC tool and used NxTrim (v0.4.3) to filter and trim adapter sequences using default parameters and a minimum length of 25bp (O’Connell *et al.* 2015). This software also sorts read pairs from mate pair libraries into one of three categories based on the presence (or absence) and the position of junction adapters in read pairs: a mate pair bin, a paired end bin, and an unknown bin. We excluded the last category of reads from the assembly because it is impossible to tell whether reads came from one side or opposite sides of the junction adapter. A fourth bin containing single end reads is produced when one read from a pair is completely trimmed. This process of trimming and sorting reads from mate pair libraries significantly improves scaffold lengths and reduce mis-assemblies (Leggett *et al.* 2013, O’Connell *et al.* 2015). While paired end reads in the unknown category and single end reads were not used for assembly, these reads were treated as single end reads and used in later error correction and genome polishing steps.

NxTrim processed reads were then subject to another round of filtering and trimming with CLC Genomics Workbench (version 11). This was done to remove read pairs that were the result of index hopping among 200bp, 5.2kb, and 10kb libraries, which were multiplexed and run on the same lanes. The 3kb and 20kb libraries were run on separate lanes so there was no potential for index hopping. The additional round of read processing with CLC Genomics Workbench also ensured that junction and sequencing adapters were completely removed and that ambiguous sequences (limit = 2 N’s) and low-quality bases (quality limit = 0.05) were trimmed. CLC Genomics Workbench uses a modified-Mott trimming algorithm, which converts Phred (Q) scores to error probabilities and uses the quality limit as a threshold to determine stretches of low quality bases (*i.e.*, high error probabilities) to be trimmed. We used CLC Genomics Workbench to filter phiX174 vector sequences and snapping turtle mitochondrial DNA sequences (mapping parameters; match score 1; mismatch cost 2; insertion and deletion cost 3; length fraction 0.96; similarity fraction 0.98). We discarded trimmed reads <25bp, but saved quality reads from broken pairs.

We assessed the empirical distribution of insert sizes for paired end and mate pair libraries by aligning reads to the initial assembly with Bowtie 2. We then calculated the mean and standard deviation of insert sizes to further refine input parameters for Allpaths-LG. Actual sizes of paired end and mate pair inserts were close to nominal sizes for all Illumina libraries.

We error-corrected PacBio reads using LoRDEC (v0.9) (Salmela and Rivals 2014), a hybrid error correction software that uses de Bruijn graphs constructed with trimmed and filtered Illumina reads. We used CANU (v1.8) (Koren *et al.* 2017) to correct and trim Nanopore sequences.

### Genome assembly and completeness

We first estimated the size of the snapping turtle genome using k-mer frequency histograms derived from short reads and BBmap software (version 38.24) (Bushnell 2014). Genome assembly was then done in three distinct steps. In the first step, we employed ALLPATHS-LG (version 52448) (Gnerre *et al.* 2011), a whole‐genome shotgun assembler. In the second step, we employed PBJelly (version 15.8.24) (English *et al.* 2012) and error-corrected PacBio reads to fill gaps and join scaffolds from the initial assembly produced by ALLPATHS-LG. After PBJelly, we used Pilon software (version 1.16) and the trimmed and filtered Illumina reads for error correction (Walker *et al.* 2014). In the third step, we used CANU (v1.8) (Koren *et al.* 2017) to produce an independent genome assembly with Nanopore sequences. We then used the intermediate assembly described above (with a very low error rate), the CANU assembly (with a higher error rate from long read technology), and *quickmerge* software (version 0.2) (Chakraborty *et al.* 2016) to further increase the contiguity of the snapping turtle genome. In brief, *quickmerge* identifies high confidence overlaps between two assemblies and joins contigs and scaffolds when overlap quality surpasses user-defined thresholds. Thresholds are based on the relative length of aligned *vs.* unaligned regions within the entire overlapping regions to minimize the potential for spurious joining of contigs/scaffolds. We used default settings for the overlap cutoffs for selection of anchor contigs (-hco = 5.0) and extension contigs (-c = 1.5). We used the scaffold N50 from the pilon corrected CANU assembly as the length cutoff for anchor contigs (-l = 1,088,418 bases). We used the default setting for minimum alignment length to be considered for merging (-ml = 5000).

The intermediate genome assembly was used as the “reference” genome, while the CANU assembly was used as the “query” genome. The *quickmerge* algorithm preferentially uses the more accurate sequence from the “reference” genome in the newly joined contigs/scaffolds, while the “query” genome is used to join together higher quality contigs/scaffolds. The final draft genome was error corrected with Pilon software (version 1.16) and trimmed and filtered Illumina reads (Walker *et al.* 2017). Completeness of the final draft genome was assessed with Benchmarking Universal Single-Copy Orthologs (BUSCO) (Simão *et al.* 2015). We used Vertebrata datasets from OrthoDB V9 database containing a total of 2,586 BUSCO groups.

### Repeat annotation

Repetitive elements in the snapping turtle genome were discerned by homology searches against known repeat databases and also by *de novo* prediction. We employed RepeatModeler (version open-1.0.11) to build a *de novo* snapping turtle repeat library (Smit and Hubley 2015). This library was subsequently used to predict, annotate and mask repeats in the snapping turtle genome using RepeatMasker (version open 4.0) (Smit *et al.* 2015). We used *LTRharvest* (GenomeTools, version 1.5.9) (Ellinghaus *et al.* 2008) for *de novo* predictions of LTR (Long Terminal Repeat) retrotransposons.

### Individual heterozygosity

Trimmed and filtered Illumina reads were mapped to the final draft genome with CLC Genomics Workbench (no masking; match score 1; mismatch cost 2; insertion cost 2; deletion cost 3; length fraction 0.98; similarity fraction 0.98). Reads were locally realigned with multi-pass realignment (3 passes). We then called variants using the “Fixed Ploidy Variant Detector” (ploidy 2; required variant probability 95%; ignore positions with coverage above 150; ignore non-specific matches; minimum coverage 20; minimum count 4; minimum frequency 20%; base quality filter default settings). We excluded variants that were called homozygous by the software. Random variation in sequencing depth across the genome and random sequencing of alleles lead to variation from the expected allele frequency of 50% in a heterozygote so we only included variants that had allele frequencies between 25% and 75% for further analysis.

### Transcriptome assembly and gene prediction

For transcriptome assembly, Illumina RNA-Seq reads (Table 7) were obtained from various tissues at different developmental stages (embryonic hypothalamus and pituitary gland; embryonic gonads; hatchling hypothalamus and pituitary gland; hatchling intestine; juvenile heart) and from dissociated embryonic gonad cells in culture. We also sequenced RNA from embryonic gonads on the Roche 454 GS-FLX platform (Table 7). RNA quantity was measured using the Quanti-iT RNA assay kit and a Qubit fluorometer. RNA purity was assessed via absorbance measurements. All RNA samples had A260/A280 ratios between 1.75 and 2.0 and A260/230 ratios between 1.5 and 2.0. RNA integrity was examined via gel electrophoresis or Agilent TapeStation. All RNA samples had distinct 18S and 28S rRNA bands with minimal evidence of degradation (RINs were greater than 8.4).

We used the FastQC tool and CLC Genomics Workbench to trim adapter sequences and low quality bases (q-score <20) from Illumina RNA-Seq reads. Trimmed and quality filtered reads were used for transcriptome assembly using several *de novo* and reference-based strategies. For *de novo* assembly, reads from all RNA-Seq libraries were assembled together using CLC Genomics Workbench (Table 8). Reference aided assembly was performed separately for each tissue type (hypothalamus/pituitary, intestine, gonad, heart, and gonadal cells) by mapping Illumina reads to our assembled genome using Tophat (v2.1.1) and Trinity assembler with default parameters (v2.8.5) (Grabherr *et al.* 2011) (Table 8). Transcripts assembled using CLC Genomics Workbench and Trinity were investigated to identify potential protein-coding transcripts using TransDecoder with a minimum open reading frame of 66 amino acids (v5.5.0) (Haas *et al.* 2013).

We also used reference-guided assembly with protein-coding transcripts from *Chrysemys picta* (ftp://ftp-ncbi.nlm.nih.gov/genomes/Chrysmemys_picta/RNA), *Alligator mississippiensis* (ftp://ftp-ncbi.nlm.nih.gov/genomes/Alligator_mississippiensis/RNA), and *Terrapene mexicana triunguis* (ftp://ftp-ncbi.nlm.nih.gov/genomes/Terrapene_mexicana_triunguis/RNA) on CLC Genomic workbench (Table 8). Reads from the snapping turtle were mapped to transcripts from each species and consensus snapping turtle transcripts were extracted.

Finally, RNA from embryonic adrenal-kidney-gonad (AKG) complexes was used for direct-cDNA sequencing on the GridION system (Oxford Nanopore Technologies). Nanopore reads from direct cDNA sequencing were error-corrected using proovread (v 2.14.0) (Hackl *et al.* 2014) and adapter sequences removed using Porechop (v0.2.4; https://github.com/rrwick/Porechop). Cleaned Nanopore reads were assembled using CLC Genomic Workbench and CANU (v1.8) (Koren *et al.* 2017) (Table 8). All together, we produced 10 transcriptome assemblies using RNA from numerous tissues and sequencing platforms, as well as different assembly algorithms.

Putative protein-coding transcripts from these 10 independent assemblies were further processed with Mikado (v1.2) using default parameters (Venturini *et al.* 2018). Mikado uses a novel algorithm to integrate information from multiple transcriptome assemblies, splice junction detection software, and homology searches of the Swiss-Prot database to select the best-supported gene models and transcripts. We ran Mikado three times to recover as many potential protein-coding genes as possible. The first run produced 134,687 gene models, the second run produced 3,085 additional gene models, and the third run produced another 946 gene models for a total of 138,718 models of putative protein-coding genes.

We then used Maker (Cantarel *et al.* 2008) to increase the accuracy of gene models, reduce redundancy of overlapping models from the Mikado gene set, and predict new gene models that Mikado may have missed. We ran Maker basic protocol 2 (version 2.31.10), which is designed to update and combine legacy annotations (*i.e.*, the Mikado gene models) in the light of new evidence (Campbell *et al.* 2014). Input for Maker included the final snapping turtle genome assembly, the 138,718 gene models from Mikado, protein evidence from the American alligator (*Alligator mississipiensis*), protein evidence from several turtle species (*i.e.*, *Chelonia mydas*, *Chrysemys picta bellii*, *Gopherus evgoodei*, *Pelodiscus sinensis*, *and Terrepene carolina triunguis*), as well as snapping turtle transcripts (*i.e.*, all 1,108,260 transcripts assembled with CLC Genomics Workbench, but not filtered with TransDecoder). Maker produced 30,166 models for putative protein-coding genes.

We assessed Mikado and Maker gene models by blasting predicted transcripts against the painted turtle (*Chrysemys picta*) proteome. Based on BLASTX hits to *Chrysemys picta* proteins, there were 15,718 protein-coding genes in common between Mikado and Maker gene sets. However, there were also differences between gene prediction software. The Mikado gene set contained hits to 1,071 *Chrysemys picta* proteins that were not in the Maker gene set (*i.e.*, Maker lost these genes). Conversely, the Maker gene set contained hits to 614 *Chrysemys picta* proteins that were not in the Mikado gene set (*i.e.*, Maker discovered these genes). This comparison revealed that Mikado and Maker each produced a significant number of gene models the other software missed. To avoid losing protein-coding genes, we used both Mikado and Maker gene models in the following pipeline.

To obtain a final set of gene models that are likely to encode real proteins, we ran predicted snapping turtle proteins from Mikado and Maker through OrthoFinder with default settings (Emms and Kelly 2019). OrthoFinder classifies proteins from two or more species into sets of proteins called “orthogroups” that contain orthologs and/or paralogs. We used proteomes from mammals (*Homo sapiens*, *Mus musculus*, and *Rattus norvegicus*), archosaurs (*Gallus gallus* and *Alligator mississippiensis*), and turtles (*Chrysemys picta*, *Pelodiscus sinensis*, *and Terrapene carolina triunguis*) to identify 49,518 snapping turtle proteins that were members of “orthogroups” with proteins from at least one other vertebrate species. We then filtered exact sequence duplicates at the mRNA level to select 43,093 gene models. We further reduced redundancy by running mRNAs through CD-Hit-EST at a 98% identity level to produce a penultimate set of 25,630 gene models for protein-coding genes.

Finally, we used bedtools (v2.27.1) to cluster overlapping gene models on the same strand and remove redundant gene models that represent alternative splice variants of the same gene (Quinlan and Hall 2010). We checked both strands for gene models and removed single exon predictions with no homology to proteins in other species. We also removed single exon predictions that contained internal stop codons. This produced a final set of 22,812 gene models for protein-coding genes in the common snapping turtle.

### Gene annotation

Many researchers simply carry out BLASTP to the Swiss-Prot database and adopt gene names and symbols from the best hit, which leads to propagation of annotation errors (Salzberg 2019). In addition, genes that are duplicated (*i.e.*, paralogs) or lost (*i.e.*, gene deletion) in different lineages or species make it difficult to accurately assign gene names/symbols to orthologs. We therefore used OrthoFinder to annotate our final set of 22,812 protein-coding genes based on orthology among several amniotic vertebrates. We first assigned human gene names and symbols to 11,835 genes that displayed one-to-one orthology across snapping turtles, humans, and at least one other species (alligator, chicken, painted turtle, or box turtle). We then assigned alligator gene names and symbols to 840 genes based on one-to-one orthology across snapping turtles, alligator, and one other species (chicken, painted turtle, or box turtle). Another 236 genes were annotated with chicken gene names and symbols based on one-to-one orthology across snapping turtles, chicken, and one other turtle (painted turtle or box turtle). A fourth set of 1376 genes was annotated based on one-to-one orthology across snapping turtle, painted turtle, and the box turtle. Gene symbols from non-human databases were converted to HUGO Gene Nomenclature Committee (HGNC) gene symbols for orthologous genes. This process produced high confidence gene names and symbols for 14,287 protein-coding genes.

We used BLASTP to assign gene names and symbols to 690 more genes that had hits to the Swiss-Prot database (when all hits had the same unique name) and to 743 more genes that had hits to box turtle proteins (when there was also supporting evidence from Swiss-Prot). Gene names and symbols were assigned to 15,720 genes. Some symbols did not meet NCBI guidelines so these were replaced with locus tags (see Eukaryotic Genome Annotation Guide; https://www.ncbi.nlm.nih.gov/genbank/eukaryotic_genome_submission_annotation/#protein_id). These genes still have unique gene names, but do not have gene symbols. Another 4,930 genes were annotated with gene names based on BLASTP hits to Swiss-Prot or to box turtle proteins (*i.e.*, these genes have locus tags, but do not have HGNC gene symbols).

### Comparative and phylogenomic analysis of protein coding genes

We used OrthoFinder (Emms and Kelly 2019) to compare 22,812 snapping turtle proteins to proteomes from 16 other vertebrate species to assess the completeness of our gene models at a genome wide scale. We also used OrthoFinder and STRIDE (Emms and Kelly 2019) to carry out phylogenomic analyses to see whether evolutionary relationships among turtles are consistent with phylogenetic trees from prior studies. We retrieved proteomes from mammals (*Homo sapiens*, *Mus musculus*, and *Rattus norvegicus*), birds (*Gallus gallus*, *Maleagris gallopavo*, and *Taeniopygia guttata*), crocodilians (*Alligator mississippiensis*, *Alligator sinensis*, and *Crocodylus porosus*), and turtles (*Chelonia mydas*, *Chrysemys picta*, *Gopherus evgoodi*, *Pelodiscus sinensis*, *Platysternon megacephalum*, and *Terrapene carolina triunguis*) (Table 11). We also downloaded the proteome for a representative fish (*Danio rerio*) as an outgroup (Table 11).

### Non-coding RNAs

Transfer RNAs (tRNAs) were predicted using tRNAscan-SE (version 2.0) (Lowe and Eddy 1997) with a score threshold of 65. Putative tRNAs that overlapped protein-coding genes were removed. Ribosomal RNAs (rRNAs) were predicted using Barrnap (Seemann and Booth 2013) with a reject threshold of 0.40. Partial or shortened rRNAs were removed. Hairpin micro-RNAs (miRNA) were predicted by aligning all hairpin and mature miRNAs sequences from miRBase (release 22) (Griffiths-Jones *et al.* 2008) to the snapping turtle genome using BLASTN (e-value < 1e-10 for hairpin sequences). This gave initial predictions for 16,169 hairpin sequences and 1,175,272 mature miRNA sequences. Sequences were clustered by genomic loci, returning 1,514 hairpin clusters and 989,989 mature miRNA clusters. We then selected 899 hairpin clusters that had complete overlap with a mature miRNA sequence. miRBase entries occurring in more than one cluster were removed and clusters containing hits to less than two species were removed. The consensus name for each cluster was chosen based on the most frequent miRNA name within the cluster. Final genomic coordinates of hairpin miRNA sequences were selected based on the lowest e-value.

### Data availability

Raw data used for genome assembly, transcriptome assembly, and the final draft genome can be found in the NCBI SRA database (SUB6351883: accession numbers SRR10270339, SRR10270340, SRR10270341, SRR10270342, SRR10270343, and SRR10270344) under BioProject PRJNA574487. Scripts are available on GitHub (https://github.com/turkrhen/snapping_turtle_genome_scripts).

## Results and Discussion

### Genome assembly and completeness

Initial assembly of the snapping turtle genome using Illumina short reads produced a genome of 2.128Gb, which is similar to the 2.20Gb predicted by BBmap. The initial assembly with ALLPATHS-LG had a total of 17,865 scaffolds (Table 2). The intermediate genome assembly that incorporated PacBio long reads (ALLPATHS-LG, PBJelly, and Pilon) had a size of 2.314Gb with 16,317 scaffolds (Table 2). The longest scaffold was 11.97Mb for the initial assembly and 12.89Mb for the intermediate assembly (Table 2). The number of contigs decreased from 235,067 to 93,330, the longest contig increased 5.2 fold, and contig N50 increased 3.3 fold. Improvements in the intermediate assembly were largely driven by gap filling, with the number of gaps decreasing to one third of the initial assembly (Table 2).

**Table 2:** Statistics for the assembled *Chelydra serpentina* draft genome

Assembly software	ALLPATHS-LG	ALLPATHS-LG + PBJELLY	ALLPATHS-LG +PBJELLY+PILON	ALLPATHS-LG +PBJELLY *+ Quickmerge* +PILON
Total Length scaffold (bp)	2128820104	2314316492	2314078856	2257723393
Longest scaffold (bp)	11970359	12886104	12890361	27238941
Longest contig (bp)	386157	2025513	2020986	10156701
Number of scaffolds	17865	16317	16317	13224
Number of contigs	235067	94182	93330	52645
Number of gaps	217202	77865	77013	39421
Scaffold N50 (bp)	1191164	1357394	1358478	5589128
Contig N50 (bp)	20648	68275	68958	871274
Gaps N50 (bp)	3590	3972	3972	3961

**Figure 2:**
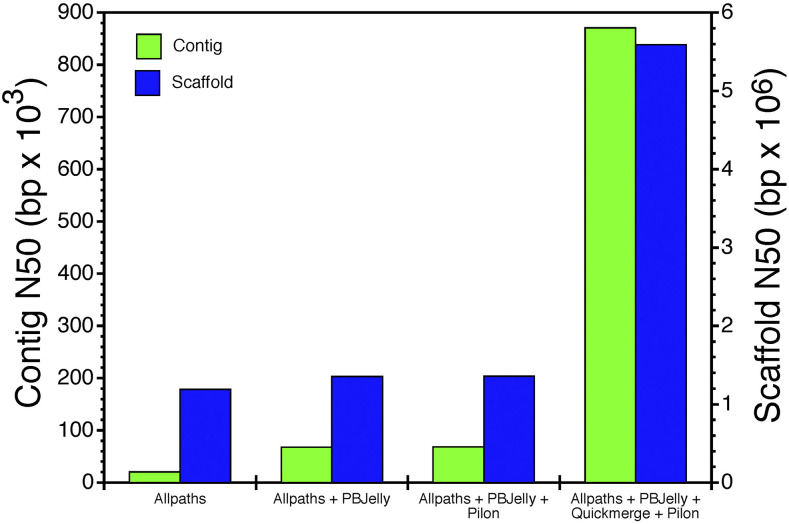
Contig and scaffold N50’s for initial, intermediate, and final assemblies of the snapping turtle genome.

Although improvements in assembly metrics were modest from the initial to the intermediate assembly, there were substantial improvements in assembly metrics with the final assembly (Table 2). For instance contig N50 and scaffold N50 increased 12.sixfold and 4.onefold, respectively (Figure 2). The final draft genome that integrated Nanopore long reads had a size of 2.258 Gb with 13,224 scaffolds (Table 2). Scaffold N50 for the final genome was 5.59 Mb while the longest scaffold was 27.24 Mb. In addition, the number of contigs and gaps dropped by half, which indicates a substantial improvement in the contiguity of the final draft genome. The GC content was estimated to be 44.34%, which is comparable to the 43–44% GC content reported in other turtle species (Shaffer *et al.* 2013, Wang *et al.* 2013, Tollis *et al.* 2017, Cao *et al.* 2019).

**Figure 3:**
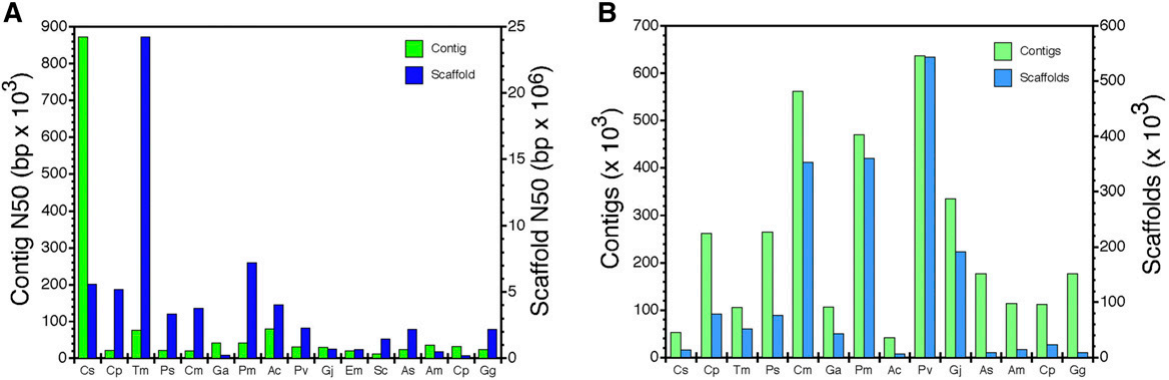
Comparison of genome assembly metrics for various reptiles. (A) Contig N50’s and scaffold N50’s and (B) number of contigs and scaffolds for *Chelydra serpentina* (Cs), *Chrysemys picta* (Cp), *Terrapene mexicana triunguis* (Tm), *Pelodiscus sinensis* (Ps), *Chelonia mydas* (Cm), *Gopherus agassizii* (Ga), *Platysternon megacephalum* (Pm), *Anolis carolinensis* (Ac), *Pogona vitticeps* (Pv), *Gekko japonicus* (Gj), *Eublepharis macularius* (Em), *Shinisaurus crocodilus* (Sc), *Alligator sinensis* (As), *Alligator mississipiensis* (Am), *Crocodylus porosus* (Cp), and *Gavialis gangeticus* (Gg).

The snapping turtle genome displays greater contiguity than most other published reptile genomes (Figure 3; Table 3). The only exception was the Mexican box turtle, which used 10X Genomics linked reads to produce a 4.3 fold longer scaffold N50 (Figure 3A; Table 3). Yet, the snapping turtle contig N50 was 11.4 fold longer than the box turtle contig N50 (Figure 3A; Table 3). The snapping turtle genome also has half as many contigs and one quarter the scaffolds as the box turtle genome (Figure 3B; Table 3). Differences in various measures of contiguity reflect the different technologies used to acquire long-range sequence information (10X Genomics linked reads in box turtle *vs.* PacBio and Nanopore long reads in snapping turtle). This suggests that linked and long reads provide complementary information that could dramatically improve genome contiguity if used together.

**Table 3:** Comparison of the *Chelydra serpentina* genome to other reptile genomes

Species	Common Name	Sequencing Technology	Coverage	Genome size (Gb)	Contig N50 (kb)	Number of Contigs	Scaffold N50 (kb)	Number of Scaffolds	Ref.
*Chelydra serpentina*	Snapping Turtle	Illumina, PacBio, Nanopore	126X	2.26	872.1	52,731	5590	13,224	
*Chrysemys picta*	Painted Turtle	Sanger, Illumina	18X	2.59	21.3	262,326	5212	78,631	1
*Terrapene mexicana*	Mexican Box Turtle	Illumina, 10X Genomics	69X	2.57	76.6	106,051	24249	52,260	NCBI
*Pelodiscus sinensis*	Chinese Softshell Turtle	Illumina	106X	2.21	21.9	265,137	3331	76,151	2
*Chelonia mydas*	Green Sea Turtle	Illumina	82X	2.24	20.4	561,968	3778	352,958	2
*Gopherus agassizii*	Desert Tortoise	Illumina	147X	2.4	42.7	106,825	251	42,911	3
*Platysternon megacephalum*	Big-headed turtle	Illumina	208.9X	2.32	41.8	470,184	7220	360,291	4
*Anolis carolinensis*	Green anole lizard	Sanger	7.1X	1.8	79.9	41,986	4033	6,645	5
*Pogona vitticeps*	Australian dragon lizard	Illumina	86X	1.77	31.2	636,524	2291	543,500	6
*Gecko japonicus*	Japanese gecko	Illumina	131X	2.49	29.6	335,470	708	191,500	7
*Eublepharis macularius*	Leopard gecko	Illumina	136X	2.02	20		664		8
*Shinisaurus crocodilus*	Chinese crocodile lizard	Illumina	149X	2.24	11.7		1470		9
*Alligator sinensis*	Chinese alligator	Illumina	109X	2.27	23.4	177,282	2188	9,317	10
*Alligator mississipiensis*	American alligator	Illumina	68X	2.17	36	114,159	509	14,645	11
*Crocodylus porusus*	Saltwater crocodile	Illumina	74X	2.12	32.7	112,407	204	23,365	11
*Gavialis gangeticus*	Gharial	Illumina	109X	2.88	23.4	177,282	2188	9,317	11

1) Shaffer *et al.* 2013, 2) Wang *et al.* 2013, 3) Tollis *et al.* 2017, 4) Cao *et al.* 2019, 5) Alföldi *et al.* 2011, 6) Georges *et al.* 2015, 7) Liu *et al.* 2015, 8) Xiong *et al.* 2016, 9) Gao *et al.* 2017, 10) Wan *et al.* 2013, 11) Green *et al.* 2014.

**Figure 4:**
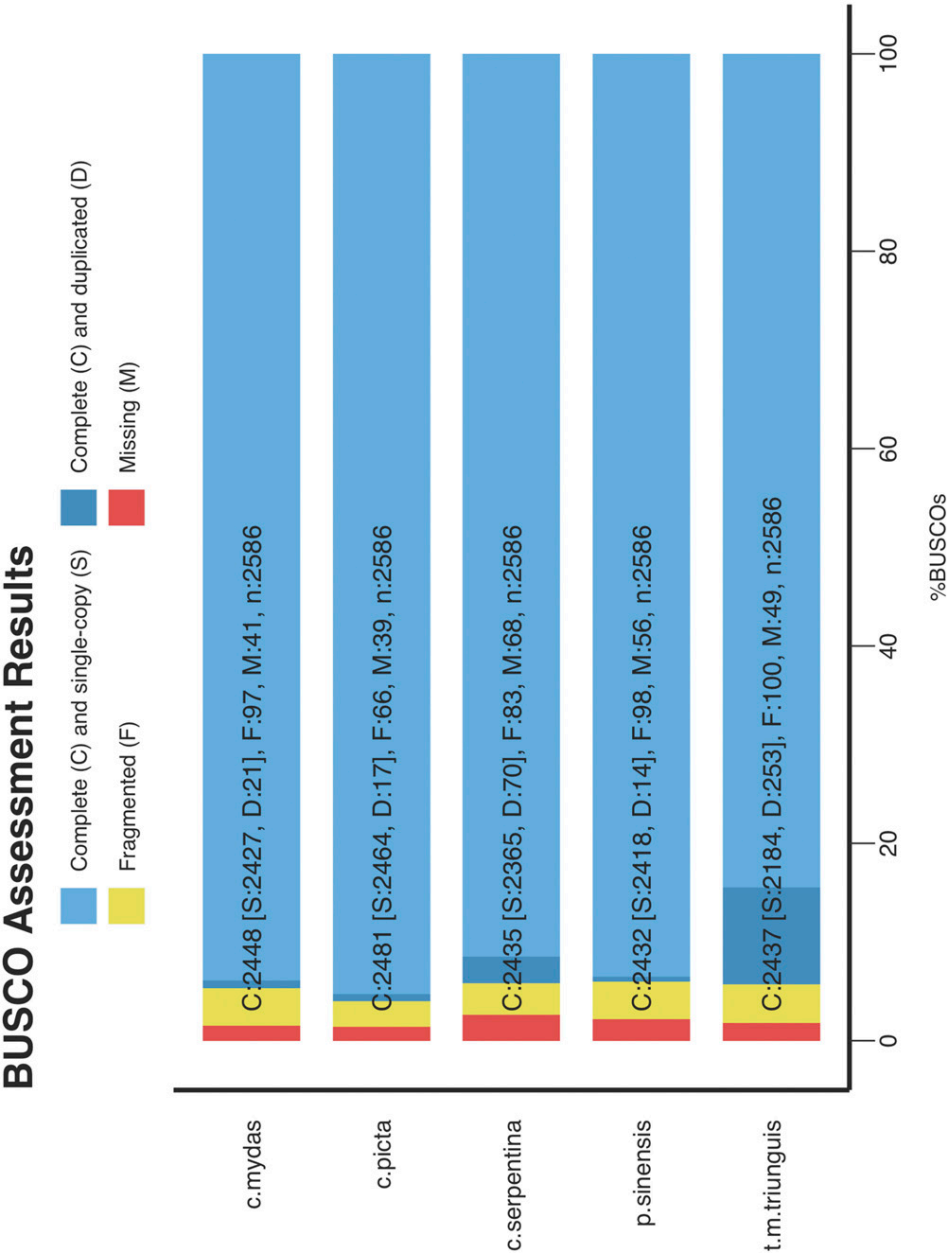
Comparison of completeness of turtle reference genomes. Genome assemblies of *Chelonia mydas*, *Chrysemys picta*, *Chelydra serpentina*, *Pelodiscus sinensis*, and *Terrapene mexicana triunguis* were compared for their completeness using BUSCO.

The snapping turtle reference genome contained both complete (94.2%) and fragmented (3.2%) core vertebrate genes as assessed via BUSCO (Table 4). This estimate of completeness is comparable to the completeness of other turtle genomes (Figure 4). Only 2.6% of the BUSCO core vertebrate genes were missing from the snapping turtle genome, which is a similar level of completeness reported in other reptiles (Gao *et al.* 2017).

**Table 4:** Summary of BUSCO analysis for the *Chelydra serpentina* draft genome

Types of BUSCOs	Count	Percentage
Complete BUSCOs	2435	94.20
Complete and single-copy BUSCOs	2365	91.50
Complete and duplicated BUSCOs	70	2.70
Fragmented BUSCOs	83	3.20
Missing BUSCOs	68	2.60

### Repetitive DNA

**Figure 5:**
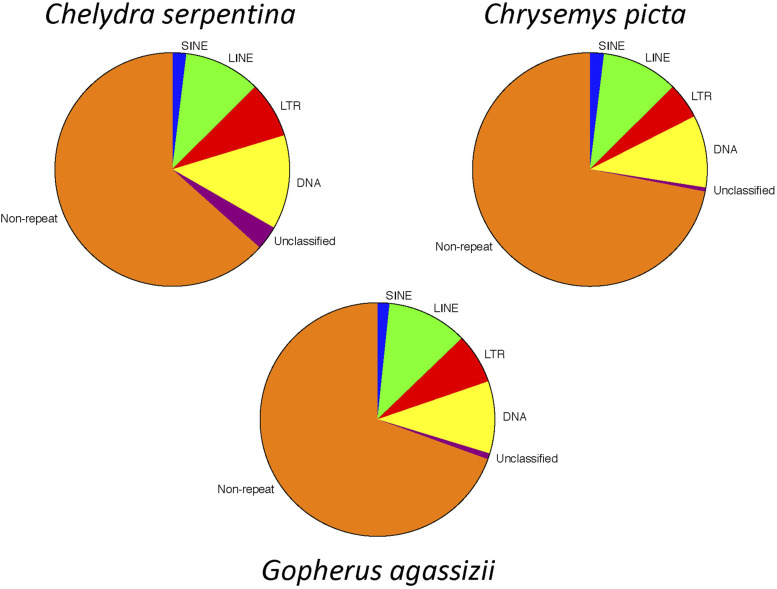
Comparison of repeat content among genomes for *Chelydra serpentina*, *Chrysemys picta*, and *Gopherus agassizii*.

The total length of repetitive elements accounted for 36.76% of the snapping turtle genome (Table 5). This is halfway between the repetitive DNA content of other turtle genomes: 29% in *Chrysemys picta* and 43% in *Gopherus agassizii* (Tollis *et al.* 2017). The greatest variation in repetitive DNA elements among species was in LTRs, DNA transposons, and unclassified repeats (Figure 5).

**Table 5:** Summary statics of interspersed repeat elements in the *Chelydra serpentina* draft genome

	Number of elements	Total Length (bp)	Percentage of sequence
SINEs:	289983	44174379	1.96
ALUs	1927	391227	0.02
MIRs	220430	31833398	1.41
LINEs:	687884	239290244	10.60
LINE1	2095	697521	0.03
LINE2	99433	18718432	0.83
L3/CR1	391880	165506806	7.33
LTR elements:	375566	176425159	7.81
ERVL	0	0	0.00
ERVL-MaLRs	0	0	0.00
ERV_classI	24667	9725298	0.43
ERV_classII	0	0	0.00
DNA elements:	1399380	291255572	12.90
hAT-Charlie	174802	39667122	1.76
TcMar-Tigger	28652	7023175	0.31
Unclassified:	222952	78727133	3.49
Total interspersed repeats		829872487	36.76

### Individual heterozygosity

A total of 3.70 million variants were detected in the reference snapping turtle, with 3.27 million single nucleotide polymorphisms (SNPs; Table 6). In comparison, 4.99 million SNPs were reported in the big-headed turtle (Cao *et al.* 2019). However, the method used to identify SNPs in the big-headed turtle was much less stringent, which explains the higher number of SNPs.

**Table 6:** Summary of genetic variants detected in the *Chelydra serpentina* draft genome (genome size = 2.314 Gb)

Variant Type	Frequency	Percentage of Variants	Variants/Mb
Small Indel	395,921	10.69%	175.4
MNP	31,435	0.85%	13.9
Replacement	6,929	0.19%	3.1
SNP	3,269,290	88.27%	1,448.0
Total	3,703,575		

**Table 7:** Summary of whole transcriptome shotgun sequence data for *Chelydra serpentina*

Tissue Type	Sequencing Platform	Library Type	Read Length	Raw Reads	Mean read length	Bases (Gb)
Embryonic and Hatchling Hypothalamus/Pituitary	Illumina	Single-end	50 bp	172244331	n/a	8.61
Embryonic Gonads	Illumina	Single-end	100 bp	153596329	n/a	15.36
Hatchling Intestine	Illumina	Single-end	50 bp	31757630	n/a	1.59
Juvenile Heart	Illumina	Paired-end	150 bp	366536144	n/a	54.98
Cultured Embryonic Gonad Cells	Illumina	Paired-end	50 bp	446985548	n/a	22.35
Embryonic Gonads	454		Variable	2255133	387 bp (151-825 bp)	0.87
Embryonic Adrenal-Kidney-Gonad Complex	Nanopore	Direct cDNA	Variable	3164253	1386 bp (101-26870 bp)	4.39
			**total =**	**1176539368**	**total =**	**108.15**

**Table 8:** Summary of intermediate transcriptome assemblies for *Chelydra serpentina*

Assembly Type	Sequencing Platform	Tissue Type	Assembler	Total Transcripts	Transdecoder Transcripts	blast2cap3	Mikado Input
Reference aided (A. mississippiensis)	Illumina & 454	H/P, G, I, H, C	CLC Genomics	35436	n/a		35436
Reference aided (C. picta)	Illumina & 454	H/P, G, I, H, C	CLC Genomics	38262	n/a		38262
Reference aided (T. carolina)	Illumina & 454	H/P, G, I, H, C	CLC Genomics	29707	n/a		29707
*De novo*	Illumina & 454	H/P, G, I, H, C	CLC Genomics	1161412	160679	154815	154815
*De novo*	Nanopore direct cDNA	AKG	Canu	11924	n/a		11924
*De novo*	Nanopore direct cDNA	AKG	CLC Genomics	9025	n/a		9025
*De novo*	Illumina	G	Trinity	382845	99801		99801
*De novo*	Illumina	H	Trinity	613600	151273		151273
*De novo*	Illumina	H/P, I	Trinity	286368	75823		75823
*De novo*	Illumina	C	Trinity	837323	124988		124988

Key to tissue types: Embryonic and Hatchling Hypothalamus/Pituitary (H/P), Embryonic Gonads (G), Hatchling Intestine (I), Juvenile Heart (H), Cultured Embryonic Gonad Cells (C), Embryonic Adrenal-Kidney-Gonad Complexes (AKG).

**Table 9:** Comparative genomic assessment of testudine, archosaur (crocodilian and bird), mammalian, and fish proteins using OrthoFinder

	Total Genes	Genes in Orthogroups	Unassigned Genes	Percentage of Genes in Orthogroups	Percentage of Unassigned Genes	Orthogroups in Species	Percentage of Orthogroups in Species	Species-specific Orthogroups	Genes in Species-specific Orthogroups	Percentage in Species-specific Orthogroups
C. serpentina	22803	22735	68	99.7	0.3	15511	65.9	30	109	0.5
C. mydas	28672	28243	429	98.5	1.5	15263	64.9	41	104	0.4
C. picta	22376	22125	251	98.9	1.1	15719	66.8	20	84	0.4
T. mexicanum	22255	22030	225	99	1	15515	66	49	110	0.5
P. megacephalum	21529	18356	3173	85.3	14.7	14691	62.5	98	239	1.1
G. evgoodei	33407	32428	979	97.1	2.9	14855	63.1	142	375	1.1
P. sinensis	18111	17556	555	96.9	3.1	13534	57.5	21	83	0.5
A. misssissippiensis	24656	19985	4671	81.1	18.9	14880	63.3	123	583	2.4
A. sinensis	43105	42637	468	98.9	1.1	15315	65.1	175	620	1.4
C. porosus	28676	28570	106	99.6	0.4	13289	56.5	26	72	0.3
G. gallus	18112	17666	446	97.5	2.5	13383	56.9	40	207	1.1
M. gallipova	29660	28664	996	96.6	3.4	13996	59.5	272	793	2.7
T. guttata	42360	41739	621	98.5	1.5	13572	57.7	269	1574	3.7
H. sapiens	20659	19860	799	96.1	3.9	15399	65.5	127	725	3.5
M. musculus	21960	21442	518	97.6	2.4	15813	67.2	99	1025	4.7
R. norvegicus	21647	21076	571	97.4	2.6	15594	66.3	81	529	2.4
D. rerio	52829	51014	1815	96.6	3.4	15703	66.8	2263	13308	25.2

**Table 10:** Functional annotation of *Chelydra serpentina* proteins based on *de novo* prediction using Interproscan and evolutionary homology to human proteins (*i.e.*, one-to-one orthologs). Total numbers are the result of merging *de novo* annotations with homology-based annotations and reducing redundant terms (*i.e.*, eliminating duplicates)

	Annotation Database	Proteins Annotated	Number of Annotations	Number of Unique Terms
Interproscan	GO	13558	34064	2499
	KEGG	1015	2800	801
	Reactome	5341	19076	1454
Homology	GO	12704	216110	17057
	KEGG	967	2622	866
	Reactome	7802	31824	1842
Total (merged)	GO	17910	234877	17169
	KEGG	1212	3365	935
	Reactome	8991	34058	1857

**Table 11:** Accession numbers for vertebrate proteomes used for comparison to the snapping turtle genome

Proteome	Database	Accession	Isoforms
Danio rerio	NCBI	GCF_000002035.6	Yes
Homo sapiens	UniProt	UP000005640	No
Rattus norvegicus	UniProt	UP000002494	No
*Mus musculus*	UniProt	UP000000589	No
Taeniopygia guttata	NCBI	GCF_008822105.2	Yes
Meleagris gallopavo	NCBI	GCF_000146605.3	Yes
Gallus gallus	UniProt	UP000000539	No
Pelodiscus sinensis	UniProt	UP000007267	No
Platysternon megacephalum	NCBI	GCA_003942145.1	Yes
Chrysemys picta	NCBI	GCF_000241765.3	No
Terrapene carolina triunguis	NCBI	GCF_002925995.2	No
Gopherus evgoodi	NCBI	GCA_002896415.1	Yes
Chelonia mydas	UniProt	UP000031443	No
Crocodylus porosus	NCBI	GCF_001723895.1	Yes
Alligator mississippiensis	UniProt	UP000050525	No
Alligator sinensis	NCBI	GCF_000455745.1	Yes

Genome-wide levels of individual heterozygosity have not yet been reported for any other turtle species so we compared the snapping turtle to mammals. We found 3.27 million SNPs in the reference snapping turtle (genome size = 2.258Gb), while studies of individual humans reported 3.07, 3.21, and 3.32 million SNPs in an Asian and two Caucasians, respectively (Levy *et al.* 2007, Wang *et al.* 2008, Wheeler *et al.* 2008). Individual heterozygosity for SNPs in the snapping turtle, after correction for the difference in genome size, is slightly higher than observed in humans. Moreover, the 1,448 heterozygous SNPs/Mb observed in the snapping turtle falls in the upper range observed in 27 mammalian species (Abascal *et al.* 2016). While population genomic studies will be required to draw firm conclusions, the relatively high level of heterozygosity in the reference snapping turtle suggests that inbreeding and/or population bottlenecks were not a common occurrence in its ancestors. The genetic variants identified here can be used as markers for studying the relationship between genotype and phenotype, as well as for analysis of genome-wide patterns of molecular evolution.

### Gene annotation

We annotated 20,650 protein-coding genes, which is very similar to the number found in the painted turtle (21,796) and desert tortoise (20,172) genomes. The remaining 2,162 models for protein-coding genes in the snapping turtle did not display homology to other known genes and are considered hypothetical proteins at this time. We assessed the accuracy of our automated annotations by conducting manual BLASTN of cDNA sequences for 2,006 gene models that were assigned HGNC gene symbols. We used GeneCards.org to crosscheck gene names/symbols that did not match manual BLASTN hits to determine whether gene names/symbols were aliases or incorrect annotations. Aliases were considered correct because they are synonyms for the same locus.

Most automated annotations with our orthology-based pipeline were correct (97.7%; n = 1960), while a small percentage (2.3%; n = 46) were incorrect. Most of the incorrectly annotated genes were assigned gene names and symbols of close paralogs (1.5%; n = 31), but some annotations were completely incorrect (0.7%; n = 15) due to propagation of annotation errors from other species. In comparison, annotation of the same genes based on the top hit to Swiss-Prot was less accurate (96.4% correct, n = 1933; 3.6% incorrect, n = 73). The rate of completely incorrect names and symbols doubled with annotation based on the top hit to Swiss-Prot (1.6%; n = 32). In addition, slightly more genes were assigned names and symbols of close paralogs rather than orthologs (2.0%; n = 41).

### Comparative analysis of protein coding genes and phylogenomic relationships

**Figure 6:**
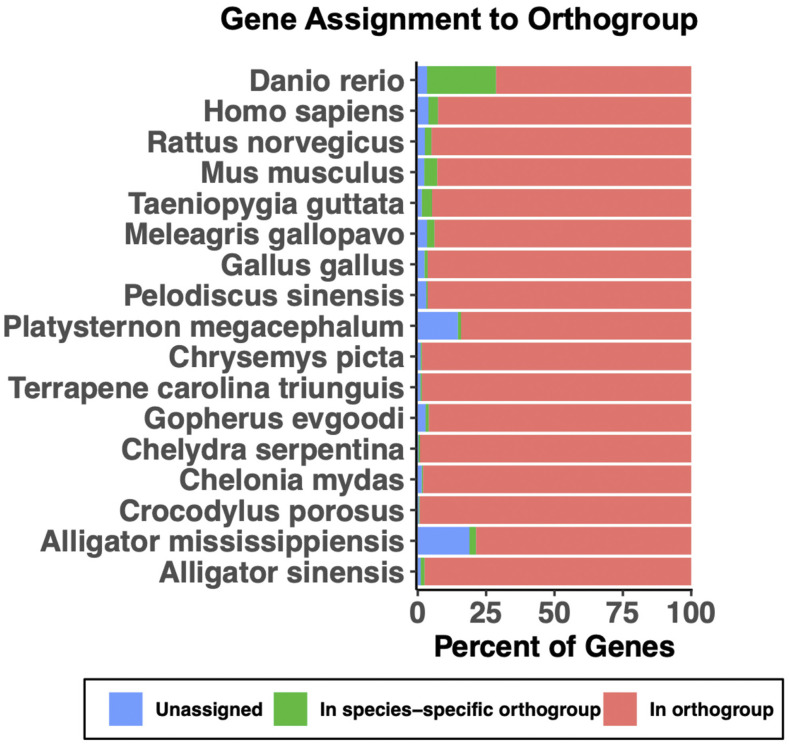
Percentage of protein coding genes assigned to orthogroups in representative vertebrate species.

The vast majority (22,735; 99.7%) of protein-coding genes in snapping turtles were assigned to orthogroups (Figure 6; Table 9), which are gene lineages comprised of orthologs and paralogs. This is similar to the number of genes assigned to orthogroups in the painted turtle and the box turtle, but higher than the number in the big-headed turtle and Chinese softshell turtle (Table 9). In contrast, many more genes were assigned to orthogroups in the green sea turtle and the desert tortoise (Table 9), which may be due to sequence redundancy in those databases.

The number of orthogroups in snapping turtles (15,511) is very similar to the number of orthogroups in painted turtle, box turtle, green sea turtle, Chinese alligator, human, mouse, rat, and zebrafish (15,263 to 15,813 orthogroups) (Table 9). The number of orthogroups is an index of the number of gene families that are conserved across vertebrates. The median number of orthogroups (15,515) in the species we examined is very close to a prior estimate of orthogroups (15,559) in tetrapods (Inoue *et al.* 2015). Based on this index, gene prediction in the snapping turtle is as complete as the best annotated turtle, crocodilian, mammalian, and fish genomes.

In contrast, big-headed turtle, desert tortoise, Chinese softshell turtle, American alligator, saltwater crocodile, and bird genomes have fewer orthogroups (13,289 to 14,880) (Table 9). This suggests gene models are incomplete (*i.e.*, missing 700 to 2,200 genes) in those species or that genes have been lost during evolution in those species. Other turtles and Chinese alligator have the typical number of orthogroups found in well-annotated mammalian and zebrafish genomes so it is more likely that gene models are incomplete in big-headed turtle, desert tortoise, Chinese softshell turtle, American alligator, and saltwater crocodile. In support of this idea, birds are known to have fewer orthogroups (∼15% less) due to poor annotation of genes in GC rich regions (Botero-Castro *et al.* 2017).

**Figure 7:**
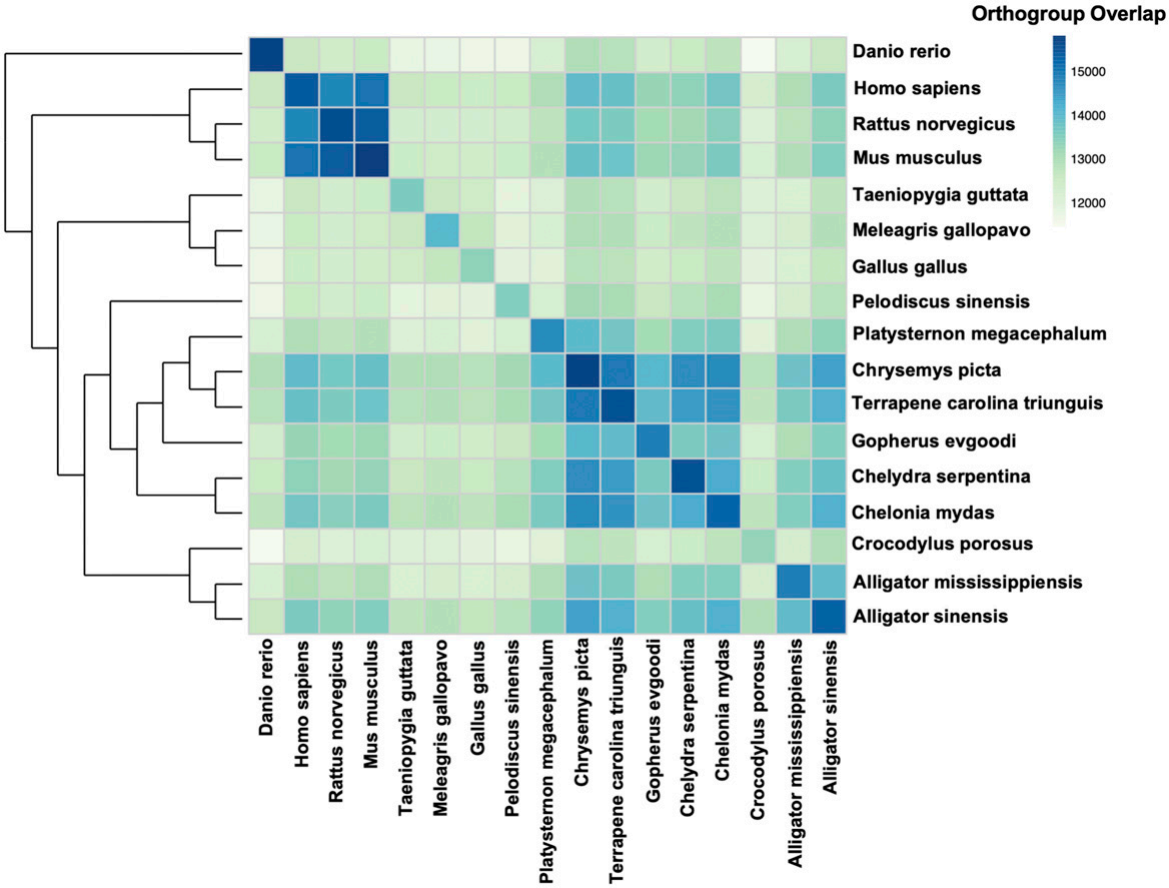
Phylogenetic relationships of common snapping turtles, other turtles, archosaurs, and mammals with complete genomes. The tree is based on analysis of orthologous genes and gene duplication events in OrthoFinder and STRIDE. The heat map represents the extent of orthogroup overlap among species, with darker colors representing more shared orthogroups and lighter colors indicating fewer shared orthogroups.

Relationships among turtles based on all protein coding genes (Figure 7) perfectly reflect phylogenetic relationships inferred from a smaller set of 539 nuclear genes (Shaffer *et al.* 2017). Snapping turtles are more closely related to sea turtles (*Chelonia mydas*) than to other turtles (Figure 7). This tree also shows the big-headed turtle is a sister species to emydid turtles and that tortoises are a sister group to both the big-headed turtle and emydid turtles. Finally, the Chinese softshell turtle is the most divergent turtle examined here. The extent of orthogroup overlap among species again suggests gene models are incomplete in the big-headed turtle, desert tortoise, Chinese softshell turtle, birds, American alligator, and saltwater crocodile (*i.e.*, lighter colors both on and off the diagonal indicate fewer shared orthogroups; Figure 7).

### Functional annotation of protein-coding genes

Experimental annotation of protein function at a genome wide scale is impractical for new model species like the snapping turtle. However, it is possible to annotate protein function based on well-characterized structural domains and by evolutionary homology to proteins in highly curated databases. In an effort to capture both conserved and divergent structural and functional elements of snapping turtle proteins we used a combinatorial approach to annotation based on structural homology to protein domains and evolutionary homology to proteins of known function. We used InterProScan (version 5.36-75.0) to assign Gene Ontology terms, KEGG pathways, and REACTOME pathways to snapping turtle proteins (Table 10). This resulted in *de novo* functional annotation of 13,558 proteins based on protein architecture and functional domains. For more complete functional annotation, we also adopted Gene Ontology terms, KEGG pathways, and REACTOME pathways associated with 12,704 genes identified as one-to-one orthologs to human genes (Table 10). We merged results from these methods and reduced redundancy of functional annotations (*i.e.*, duplicate terms). This resulted in a large set of annotations inferred from both protein signatures and evolutionary homology. As such, they should be viewed as putative rather than definitive annotations.

### Non-coding RNAs

tRNAscan-SE predicted a total of 687 tRNAs and Barrnap predicted 43 rRNAs in the snapping turtle genome. Alignment and filtering of known hairpin and mature micro-RNAs (miRNA) sequences from miRBase returned a set of 204 high confidence hairpin miRNA sequences in the snapping turtle genome.

### Summary assessment of genome assembly and annotation

Here we describe *de novo* assembly and annotation of the snapping turtle genome using both short and long read sequencing technologies and several genome assembly algorithms. The contiguity of this assembly (contig N50, scaffold N50, and number of contigs/scaffolds) is greater than most other published turtle and reptile genomes (Table 3) (Alföldi *et al.* 2011, Shaffer *et al.* 2013, Wan *et al.* 2013, Wang *et al.* 2013, Green *et al.* 2014, Georges *et al.* 2015, Liu *et al.* 2015, Xiong *et al.* 2016, Gao *et al.* 2017, Tollis *et al.* 2017, Cao *et al.* 2019). Gene and repeat content in the snapping turtle is very similar to other turtles. We provide the first assessment of individual heterozygosity at a genome-wide scale in a turtle and find it is at the upper end of the range of heterozygosity observed in mammals. This observation is consistent with the broad geographic range and abundance of snapping turtles across North America. The reference genome and genetic variants identified here provide a foundation for molecular genetic, quantitative genetic, and population genomic studies of adaptation to climate in the snapping turtle. An abundant species like the snapping turtle serves as a tractable model to identify specific genes underlying genome-environment interactions. Of particular interest are genes that influence thermosensitive sex determination, which can then be studied in threatened and endangered turtle species.
